# ANALYSIS OF THE PATTERN AND MECHANISM OF ELBOW INJURIES RELATED TO ARMBAR-TYPE ARMLOCKS IN JIU-JITSU FIGHTERS

**DOI:** 10.1590/1413-785220172505171198

**Published:** 2017

**Authors:** THIAGO BERNARDO CARVALHO DE ALMEIDA, EIFFEL TSUYOSHI DOBASHI, ALEXANDRE YUKIO NISHIMI, EDUARDO BERNARDO DE ALMEIDA, LUCIANO PASCARELLI, LUCIANO MILLER REIS RODRIGUES

**Affiliations:** 1. Department of Orthopedics and Traumatology, Escola Paulista de Medicina, Universidade Federal de São Paulo, São Paulo, SP, Brazil.; 2. Shoulder and Elbow Surgery Group, Hospital IFOR- Rede D`Or, São Bernardo do Campo, SP, Brazil.; 3. FMABC - Faculdade de Medicina ABC, Santo André, SP, Brazil.

**Keywords:** Elbow, Elbow joint, Dislocations, Athletic injuries

## Abstract

**Objective:**

The objective of this study was to analyze elbow injuries and their probable mechanism in Jiu-Jitsu fighters resulting from the armbar-type armlock.

**Methods:**

We evaluated 5 high-performance Jiu-Jitsu fighters from the Gracie Elite gym who were injured during a tournament. All were healthy males with a mean age of 28.8 years. The right arm was involved in three patients (60%). The athletes were followed for approximately 4.6 months, and pain was present in all cases. Clinical examination of the elbow was performed immediately after the injury and when magnetic resonance imaging (MRI) was performed. The radiography showed no changes. Clinical examination detected specific tender points on the medial and anterior topography of the elbows, but no ligamentous instability of the elbow was seen during dynamic testing.

**Results:**

The main MRI findings were injury to the common flexor tendon and the ulnar collateral ligament, bone contusion of the distal humerus and olecranon, and joint effusion.

**Conclusion:**

The main pattern of injury indicated by the MRI in the athletes was injury to the medial elbow complex. The primary mechanism that determined the injury was most likely elbow hyperextension applied with the forearm in neutral position of forearm. **Level of Evidence IV, Case Series.**

## RESUMO

**Objetivo:**

Demonstrar os resultados da análise das lesões do cotovelo e seu provável mecanismo em cinco atletas lutadores de jiu-jítsu decorrentes da chave de braço do tipo armlock.

**Métodos:**

Foram avaliados cinco lutadores de jiu-jítsu da academia Gracie Elite, de alto rendimento esportivo, que sofreram lesão durante a realização de um campeonato dessa modalidade. Todos eram do sexo masculino, com média de idade 28,8 anos, hígidos que sofreram lesão durante a participação nesse torneio. O braço direito foi acometido em três pacientes (60%). O seguimento dos atletas foi, em média, de 4,6 meses, sendo que a queixa de dor estava presente em todos os casos. O exame clínico da região do cotovelo foi realizado imediatamente após a ocorrência da lesão e no momento da realização do exame de ressonância magnética (RM). O exame radiográfico não demonstrou alterações. Durante o exame clínico, foram detectados pontos dolorosos específicos na topografia medial e anterior dos cotovelos examinados, na qual não se observaram instabilidades ligamentares do cotovelo durante os testes dinâmicos.

**Resultados:**

Os principais achados da RM foram: lesão do tendão comum dos músculos flexores e do ligamento colateral ulnar, contusão óssea na porção distal do úmero e do olécrano e derrame articular.

**Conclusão:**

O principal padrão de lesão reconhecido pela RM nos atletas estudados foi a lesão do complexo medial do cotovelo. Sugerimos que o mecanismo primário que determinou as lesões foi a hiperextensão do cotovelo aplicada com a posição neutra do antebraço. **Nível de Evidência IV, Série de Casos.**

## INTRODUCTION

The elbow is one of the most stable joints in the locomotor apparatus. When the architecture of this segment is disrupted due to damage to one or more of the structures, especially when associated with dislocation, there is an exacerbated chance of recurring instability, which inevitably leads to early degenerative osteoarthritis.¹

Isolated dislocation of the elbow is the second most common arm injury and is classified as simple or complex according to the presence of associated fractures; peak incidence occurs between 5 and 25 years of age.^1^ ^,^ ^2^

There is a clear perception of evolution in treatment for an unstable elbow, particularly in recent years. Studies published recently have demonstrated the anatomical and functional characteristics of the stabilizers of this structure, and have been reinforced by the insertion of the physiopathology of instability.²

The strength of the elbow is promoted by stabilizing structures where the ulnar-humeral joint, the anterior band of the medial collateral ligament (MCL), and the complex lateral ligament are the three main static elements. The periarticular muscle groups are dynamic contributors that promote an increase in constrictive force.^2^ ^,^ ^3^

According to O’Driscoll et al.,^4^ most elbow dislocations result from falls where the hand is extended. Axial force applied in valgus associated with supination is the main determining factor for damage when this force is directly transmitted on the elbow. This combination of forces produces a sequential rupture across the soft tissues that begins in the region of the lateral collateral ligament (LCL). The same force progresses to the anterior and posterior capsule and finally dissipates to the MCL.^4^ This sequence of damage determines varying levels of instability that range from partial to complete dislocation and may occasionally be associated with fractures.

The reports by Ring and Jupiter^5^ called attention to the fact that the structures responsible for the stability of the elbow are arranged as a supporting ring containing anterior, posterior, medial, and lateral elements where the possibility of instability is proportional to the gravity of the injuries. In this way, these authors corroborated the concepts presented by other previous studies.

Treatment in these cases is preferably conservative. Immobilization with a cast followed by early active mobilization is recommended to resolve the high risk of joint stiffness with concomitant limitation of range of motion. When reduction of the dislocation is easily obtained with indisputable stability, some physicians prefer not to immobilize. However, detection of joint instability is considered a criterion for repair or reconstruction of the injured ligaments.^2^ ^,^ ^6^ ^,^ ^7^

We note that Jiu-Jitsu is gradually becoming more popular and that interest in understanding and treating injuries associated with this sport has grown among physicians who work in sports medicine. When we refer to the straight armbar (also known as *juji-gatame*), we note that this type of armlock is commonly and effectively applied in fighting sports; technically, it is applied to the hyperextended elbow with the forearm kept in a neutral position. There are very few reports in the literature on injuries and the mechanism of injury in this maneuver, which determines a specific pattern of injury. As a result, this injury has increased considerably as the popularity of this sport increases. Therefore, the primary objective of this study was to describe injuries resulting from application of the straight armbar-type armlock in high-yield Jiu-Jitsu athletes.

## MATERIALS AND METHODS 

Initially, this research project was submitted to the institutional review board, as determined by Resolution 196/96 of the National Health Council for research involving humans, and was approved for implementation under CAS protocol 645458.

The participants were informed of the objectives of the study in detail and all procedures to which they would be subjected. They agreed to participate in this study and subsequently signed the informed consent form.

We considered the following inclusion criteria healthy Jiu-Jitsu athletes without comorbidities, without prior injury, without previous treatment (clinical or surgical) who injured their elbows as a result of the armbar-type armlock.

**Figure 1:**
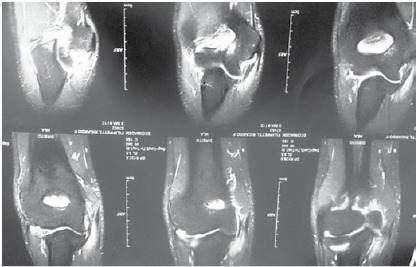
Injury to the medial complex of the elbow.

During this maneuver, the opponent is trapped in dorsal decubitus position, and the attacked arm is maintained in a neutral position between the legs of the attacking fighter. Force is then applied with the hip to hyperextend the opponent’s elbow, with the forearm maintained in neutral position. (Figure 1)

The exclusion criteria were age <18 years, cognitive or other deficit that might interfere with data collection and assessment.

Five Brazilian Jiu-Jitsu practitioners from the Gracie Elite gym who were injured as a result of the armbar-type armlock during a competition were included in the study. The injuries occurred between November 2014 and July 2015. All the patients were male, and mean age was 28.8 years. They had no comorbidities. The right elbow was affected in 60% of cases. Pain was present in 100% of cases, and the mean follow-up time averaged 4.6 months. (Table 1)

**Table 1:** Clinical data for the patients injured during armbar-type armlock of the elbow.

Patient	Age	Sex	Elbow affected	Time MRI
1	33 years	Male	Right	2 days
2	40 years	Male	Right	2 days
3	25 years	Male	Right	2 days
4	28 years	Male	Left	7 days
5	18 years	Male	Left	2 days

All the injuries were detected by the lead investigator of this study, who was also a member of the medical support staff for the tournament. This physician is also a regular practitioner of this martial art and witnessed the injuries at the exact moment when they occurred. All patients received a physical orthopedic examination in which clinical maneuvers were applied to evaluate instabilities and distinguish potentially injured structures immediately after the injury. Next, X-rays were taken of the elbows in the antero-posterior and lateral views.

The patients then underwent MR imaging, on average three days after the injury; four (80%) received MRI on the second day and one (20%) on the seventh day.

The X-ray and MR images were evaluated jointly with a team of radiologists (associated with the Brazilian College of Radiology and Diagnostic Imaging), a specialist in locomotor disorders, and three orthopedic physicians specializing in shoulder and elbow surgery (members of Brazilian Society of Orthopedics and Traumatology and the Brazilian Society for Shoulder and Elbow Surgery). 

## RESULTS

The initial clinical examination showed the following symptoms: pain in the region of the olecranon associated with diffuse pain, mainly in the medial region of the elbow and pain on palpation. Instability tests were negative.

**Figure 2:**
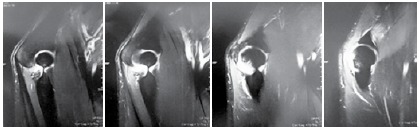
Edema of the olecranon.

X-rays of the elbow showed no changes. The MRIs in the five athletes who participated in the study showed total or partial rupture of the common flexor tendon. The ulnar collateral ligament was ruptured in 100% of the cases. (Figure 2)

**Figure 3:**
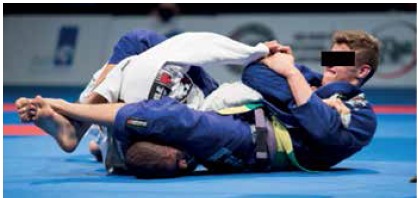
Armbar-type armlock.

We observed the presence of areas of contusion and microfractures of the bone marrow in the distal portion of the humerus and olecranon in 60% of cases. (Figure 3)

## DISCUSSION

The armbar-type armlock is one of the maneuvers most frequently applied by martial arts athletes, especially Brazilian Jiu-Jitsu. In this armlock, the attacking fighter traps his opponent in dorsal decubitus, holding the defending athlete’s arm in a neutral position between the attacking fighter’s legs. Next, force is applied with the hip to hyperextend the opponent’s elbow with the forearm in neutral rotation. We emphasize that this technique requires precise application to be effective. The defending athlete can extricate himself if he can change the position of his forearm, keeping it in a pronated or supinated position.

As for the mechanism of injury, we found no studies that biomechanically tested this fighting position. However, in the literature we found a study by An et al.,^8^ who reported that this injury entails a pattern of damage determined by the action of the muscle on the static elbow with the forearm maintained in neutral rotation; these authors evaluated the mechanical effects of this segment in positions of flexion, extension, and semi-flexion. In all positions analyzed, these authors observed that the main stabilizer of the elbow is the extensor carpi radialis, but in semi-flexion there is also a collaboration of the flexor muscles.^8^

We therefore surmise that the likely pattern of injury is caused by an eccentric force of contraction by the forearm flexor muscles, thus generating injury to the dynamic and static medial stabilizers of the elbow, in agreement with the clinical and MRI findings in our study.

## CONCLUSION

The armbar-type armlock results in an injury to the medial structures of the elbow that suffer progressively from an overload resulting in turn from a hyperextension mechanism in which the forearm is trapped in the neutral rotation position, according to the findings in our study.

We did not find similar studies in our search of the literature, indicating the originality of our publication. The active search for the injury pattern presented in accordance with the mechanism described above may assist in predicting and planning treatment in similar cases. However, there is a clear need to increase the study sample to enhance the consistency of the epidemiological findings.
